# A comprehensive analysis and comparative study of the trends in thyroid cancer burden in China and globally from 1990 to 2021, with projections for the next 15 Years

**DOI:** 10.3389/fonc.2025.1505728

**Published:** 2025-02-07

**Authors:** Yulai Yin, Xiaoyu Zhang

**Affiliations:** ^1^ Cangzhou Central Hospital Affiliated to Hebei Medical University, Cangzhou, China; ^2^ Department of Thyroid and Breast Surgery III, Cangzhou Central Hospital, Cangzhou, China

**Keywords:** thyroid cancer, disease burden, incidence, prevalence, trend

## Abstract

**Objective:**

This study aims to analyze the trends in incidence, prevalence, mortality, and disability-adjusted life years (DALYs) of thyroid cancer across gender and age groups in China and globally from 1990 to 2021, using data from the Global Burden of Disease (GBD) database. Additionally, it projects the trends in thyroid cancer burden for the next 15 years for both China and the global population.

**Materials and methods:**

Thyroid cancer-related data were extracted from the 2021 GBD dataset. The average annual percentage change (AAPC) and the corresponding 95% confidence intervals (95% CI) were calculated using Joinpoint regression to reflect trends in the thyroid cancer burden. R software was used to perform a gender- and age-specific analysis and visualize the trends in thyroid cancer burden for both China and the global population. Furthermore, the Autoregressive Integrated Moving Average (ARIMA) model was employed to project the trends in thyroid cancer burden over the next 15 years.

**Results:**

The results indicate a rising trend in the incidence and prevalence of thyroid cancer both in China and globally. Conversely, the mortality rate and DALYs show a declining trend over the same period. Age-specific analysis revealed that thyroid cancer is most prevalent among individuals aged 50–64. Gender-specific analysis indicated that the incidence rate is higher in females than in males. Projections for the next 15 years show that the age-standardized incidence rates for both males and females are expected to continue rising in China and globally. While the age-standardized mortality rate for females is projected to decline significantly, the mortality rate for males is predicted to stabilize.

**Conclusion:**

Between 1990 and 2021, the number of thyroid cancer cases has increased both in China and globally, while the mortality rate has shown a marked decline. This trend is expected to persist over the next 15 years. The growing population affected by thyroid cancer reflects a substantial disease burden, making thyroid cancer a significant global public health concern. The formulation of proactive and effective health policies is urgently needed.

## Graphical Abstract

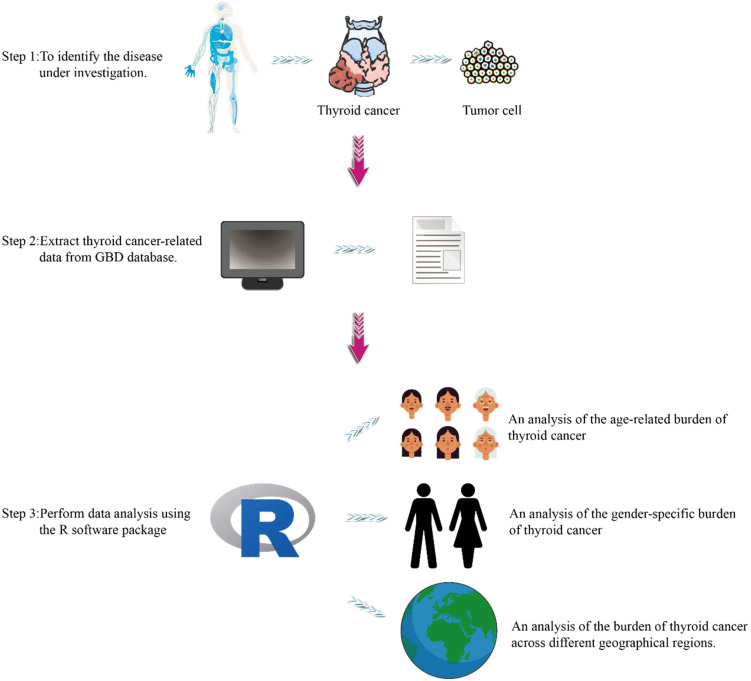

## 1 Introduction

Thyroid cancer has become one of the fastest-growing malignancies worldwide (1, 2). According to the GLOBOCAN 2020 data (3), the global age-standardized incidence rate (ASIR) for thyroid cancer in 2020 was 10.1 per 100,000 females and 3.1 per 100,000 males, with age-standardized mortality rates (ASMR) of 0.5 per 100,000 females and 0.3 per 100,000 males. The incidence rates in countries with high and very high Human Development Index (HDI) are five times higher than in low- and medium-HDI countries, while mortality rates remain relatively similar across regions (4). Over the past few decades, the incidence of thyroid cancer has risen significantly. The most recent estimates from the 2021 Global Burden of Disease (GBD) database (5) indicate a continued increase in the global incidence and mortality rates of thyroid cancer from 1990 to 2021. In China, thyroid cancer has become one of the most common endocrine malignancies, with a marked increase in the ASIR among females. This worrying trend can be partially attributed to advances in diagnostic techniques, environmental changes, and shifts in lifestyle (6, 7), which together have reshaped the epidemiological landscape.

Although thyroid cancer patients generally have a favorable prognosis, the rising incidence poses a substantial burden on public health systems. The extended treatment duration (8, 9), frequent follow-up demands, and increasing survival rates have all contributed to greater pressure on healthcare resources. Consequently, thyroid cancer has become a significant factor in driving up healthcare costs and complicating resource allocation, particularly in regions experiencing rapid urbanization and healthcare challenges (10). The combination of high incidence rates and the demand for long-term care underscores the urgent need for effective prevention, early diagnosis, and efficient management strategies (11, 12).

Current research on thyroid cancer primarily focuses on early detection technologies and the development of more targeted treatments (13). However, inconsistencies in data availability across regions limit the scope of global studies, leading to substantial regional differences in understanding the burden of thyroid cancer. Furthermore, the lack of a unified global prevention strategy underscores the need for comprehensive epidemiological studies in this area. A systematic analysis based on the GBD 2021 database provides an updated epidemiological perspective on the burden of thyroid cancer in China and globally, supporting the formulation of evidence-based policies.

The GBD database, as a scientifically rigorous data platform, offers a standardized approach to accurate assessments of incidence, prevalence, mortality, and disability-adjusted life years (DALYs) (2). By leveraging this database, researchers can conduct comparative analyses of disease burdens across regions and time periods, gaining deeper insights into the key factors driving the global rise in thyroid cancer. Additionally, the GBD methodology allows for the evaluation of the effectiveness of policies and interventions over time, providing critical scientific evidence for optimizing resource allocation and guiding future policymaking.

This study utilizes data from the GBD 2021 edition to systematically analyze the trends and burden of thyroid cancer in China and globally from 1990 to 2021. Our goal is to provide reliable data to inform public health policies, improve resource distribution, and advance strategies for the prevention and management of thyroid cancer.

## 2 Materials and methods

### 2.1 Data source

The data used in this study were obtained from the Global Burden of Disease (GBD) 2021 database (https://ghdx.healthdata.org/gbd-2021), a comprehensive dataset documenting the incidence, prevalence, and mortality of 369 diseases and injuries across 204 countries and territories, categorized by age and sex. This study extracted data related to thyroid cancer from 1990 to 2021 for China and the global population, including age-standardized incidence, prevalence, mortality, and DALY rates, differentiated by sex and age groups.

### 2.2 Statistical analysis

The data on thyroid cancer incidence, prevalence, mortality, and DALYs were extracted from the GBD database for China and globally, along with the respective age-standardized incidence rate (ASIR), age-standardized prevalence rate (ASPR), age-standardized mortality rate (ASMR), and age-standardized DALY rate (ASDR). Crude incidence rate (CIR), crude prevalence rate (CPR), crude mortality rate (CMR), and crude DALY rate (CDR) for various age groups were also extracted. The Average Annual Percentage Change (AAPC) and corresponding 95% confidence intervals (95% CI) were calculated using Joinpoint software (National Cancer Institute, Rockville, MD, USA) to assess trends in the burden of thyroid cancer. The regression model used to fit the age-standardized indicators was ln(y) = α + βx + ϵ, where y represents the age-standardized indicator and x represents the calendar year. AAPC was calculated as 100 × exp(β) − 1, with 95% CI computed from the model. If the 95% CI for AAPC is > 0, the indicator shows an increasing trend; if < 0, it shows a decreasing trend; and if equal to 0, the trend is stable.

To predict the future burden of thyroid cancer in China and globally for the next 15 years, an Autoregressive Integrated Moving Average (ARIMA) model (14) was employed. The ARIMA model, comprising autoregressive (AR) and moving average (MA) components, assumes that the data series is a stationary random variable, with its autocorrelation described by the ARIMA model, which uses historical data to forecast future trends. The model equation is Y_t = φ_1 Y_(t-1) + φ_2 Y_(t-2) + ⋯ + φ_p Y_(t-p) + e_t - θ_1 e_(t-1) - ⋯ - θ_q e_(t-q), where (φ_1 Y_(t-1) + φ_2 Y_(t-2) + ⋯ + φ_p Y_(t-p) + e_t) represents the AR component, and (e_t - θ_1 e_(t-1) - ⋯ - θ_q e_(t-q)) represents the MA component. Y_(t-p) is the observation at time (t-p), p and q are the orders of the AR and MA models, respectively, and e_t is the random error at time t. The time series must be a stationary random process with zero mean to meet the ARIMA model requirements.

In this study, the ARIMA model was employed to forecast the thyroid cancer disease burden in China and globally over the next 15 years. Given the observed trends in the incidence of thyroid cancer worldwide, the ARIMA model enables the modeling of autocorrelations within historical data, thereby capturing the evolving disease burden across different regions (e.g., China vs. global trends) and predicting future disease burdens. Specifically, we incorporated disease burden indicators stratified by age group, gender, and region, using the ARIMA model to forecast how these factors will evolve over the next 15 years, with the aim of providing data-driven support for public health policy formulation. It is important to note that, although the ARIMA model is a robust forecasting tool, it relies on the assumption that historical patterns will continue. Any significant shifts in underlying factors—such as major public health events—may impact the accuracy of the model’s predictions.

Overall, by integrating autoregression, moving averages, and differencing, the ARIMA model effectively captures dynamic variations within time series data, facilitating short- to medium-term forecasting. In this study, the ARIMA model provides a scientific basis for forecasting the future disease burden of thyroid cancer, offering valuable insights that can assist public health decision-makers in developing appropriate prevention and control strategies.

Additionally, we performed a series of validation steps for the ARIMA model. Stationarity Assumption: ARIMA models assume that the data series is stationary, meaning that the mean and variance of the data remain consistent across different time intervals. If the data exhibits trends or seasonal variations, the predictive performance of the ARIMA model may be compromised. To test for stationarity, we conducted a unit root test (ADF test) on the raw data and applied differencing to eliminate trends and non-stationarity. Furthermore, we used autocorrelation function (ACF) and partial autocorrelation function (PACF) plots to further confirm the stationarity of the data. Randomness Test: To ensure that the residuals did not exhibit significant autocorrelation, we conducted a Ljung-Box test on the model residuals and analyzed the residual ACF plots. The results showed that the residual sequence did not exhibit significant autocorrelation, indicating that the model’s assumption of randomness was satisfied. Model Selection and Parameter Optimization: We used the Akaike Information Criterion (AIC) and the Bayesian Information Criterion (BIC) as criteria for model selection to ensure that the parameters of the ARIMA model were optimal. For each data series, we iteratively tested different parameter combinations and selected the best model for forecasting. Model Robustness Verification: To further validate the predictive performance of the ARIMA model, we also introduced the Seasonal ARIMA (SARIMA) model for comparison, particularly for data with seasonal variations, to enhance forecasting accuracy. Additionally, we considered employing machine learning models, such as Long Short-Term Memory (LSTM) networks, to verify the results, ensuring that the predictions from different models were consistent and robust. This comprehensive approach was aimed at improving the reliability and precision of our forecasted outcomes.

All statistical analyses and visualizations were performed using R software (version 4.3.2) and Joinpoint software (version 4.9.1.0) (15, 16). A p-value < 0.05 was considered statistically significant.

### 2.3 Data disclosure statement

The data used in this study were sourced from the GBD 2021 database (https://ghdx.healthdata.org/gbd-2021), which does not contain any personally identifiable information. All original studies have been reviewed and approved by the relevant ethics committees, and thus no additional ethical approval was required for this study.

## 3 Results

### 3.1 Description of thyroid cancer burden in China and globally

From 1990 to 2021, the age-standardized incidence rate (ASIR) of thyroid cancer in China increased from 1.249 per 100,000 to 2.473 per 100,000, while the global ASIR rose from 2.062 per 100,000 to 2.914 per 100,000. The age-standardized prevalence rate (ASPR) in China surged from 8.098 per 100,000 to 20.012 per 100,000, and globally, the ASPR increased from 14.931 per 100,000 to 23.143 per 100,000. China’s age-standardized mortality rate (ASMR) decreased from 0.473 per 100,000 to 0.387 per 100,000, whereas the global ASMR declined from 0.570 per 100,000 to 0.530 per 100,000. The age-standardized disability-adjusted life year (DALY) rate (ASDR) in China dropped from 12.086 per 100,000 to 10.105 per 100,000, while the global ASDR decreased from 15.206 per 100,000 to 14.571 per 100,000.

The average annual percentage changes (AAPC) for ASIR, ASPR, ASMR, and ASDR in China were 2.2%, 3.0%, -0.7%, and -0.6%, respectively. In comparison, the global AAPC for these indicators were 1.1%, 1.4%, -0.2%, and -0.1%, respectively. Detailed results are presented in **Table 1**.

**Table 1:** Total number of thyroid cancer cases, Age-Standardized Incidence Rate (ASIR), Prevalence Rate (ASPR), Mortality Rate (ASMR), Disability-Adjusted Life Years (DALY) Rate, and Corresponding Average Annual Percentage Change (AAPC) in China and Globally, 1990 and 2021.

Location	Measure	1990		2021		1990-2021 AAPC
		All-ages cases	Age-standardized rates per 100,000 people	All-ages cases	Age-standardized rates per 100,000 people	
		n (95%CI)	n (95%CI)	n (95%CI)	n (95%CI)	n (95%CI)
China	Incidence	12157 (9714-14406)	1.249 (1.009-1.473)	48105 (38695-60068)	2.473 (1.993-3.088)	2.2 (2.1 - 2.4)
	Prevalence	87082 (68622-104169)	8.098 (6.41-9.66)	388411 (311967-488388)	20.012 (16.135-25.228)	3 (2.8 - 3.1)
	Deaths	3599 (3038-4182)	0.473 (0.403-0.55)	7692 (6123-9429)	0.387 (0.307-0.472)	-0.7 (-0.8 - -0.5)
	DALYS	110736 (92143-130509)	12.086 (10.142-14.08)	203325 (163131-251789)	10.105 (8.139-12.447)	-0.6 (-0.8 - -0.4)
Global	Incidence	89885 (84681-96999)	2.062 (1.951-2.224)	249538 (223290-274638)	2.914 (2.607-3.213)	1.1 (1 - 1.2)
	Prevalence	676649 (636789-727723)	14.931 (14.124-16.029)	1987148 (1776275-2198245)	23.143 (20.663-25.647)	1.4 (1.3 - 1.5)
	Deaths	21893 (20437-24108)	0.57 (0.53-0.628)	44799 (39925-48541)	0.53 (0.47-0.575)	-0.2 (-0.3 - -0.2)
	DALYS	646741 (599119-717357)	15.206 (14.184-16.83)	1246485 (1094416-1375853)	14.571 (12.783-16.115)	-0.1 (-0.2 - 0)

### 3.2 Joinpoint regression analysis of thyroid cancer burden in China and globally

**Figure 1:**
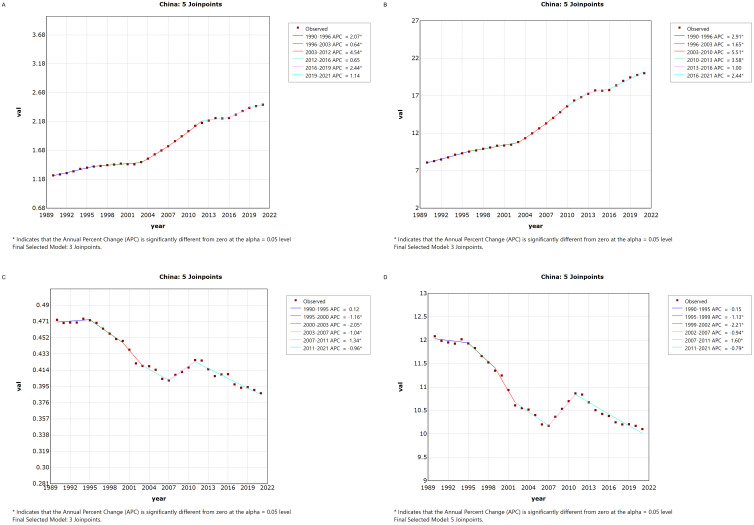
Annual Percentage Change (APC) in Age-Standardized Incidence Rate (ASIR), Age-Standardized Prevalence Rate (ASPR), Age-Standardized Mortality Rate (ASMR), and Age-Standardized Disability-Adjusted Life Year Rate (ASDR) for Thyroid Cancer in China from 1990 to 2021 (* indicates p < 0.05, denoting significant results). **(A)** ASIR; **(B)** ASPR; **(C)** ASMR; **(D)** ASDR.

**Figure 2:**
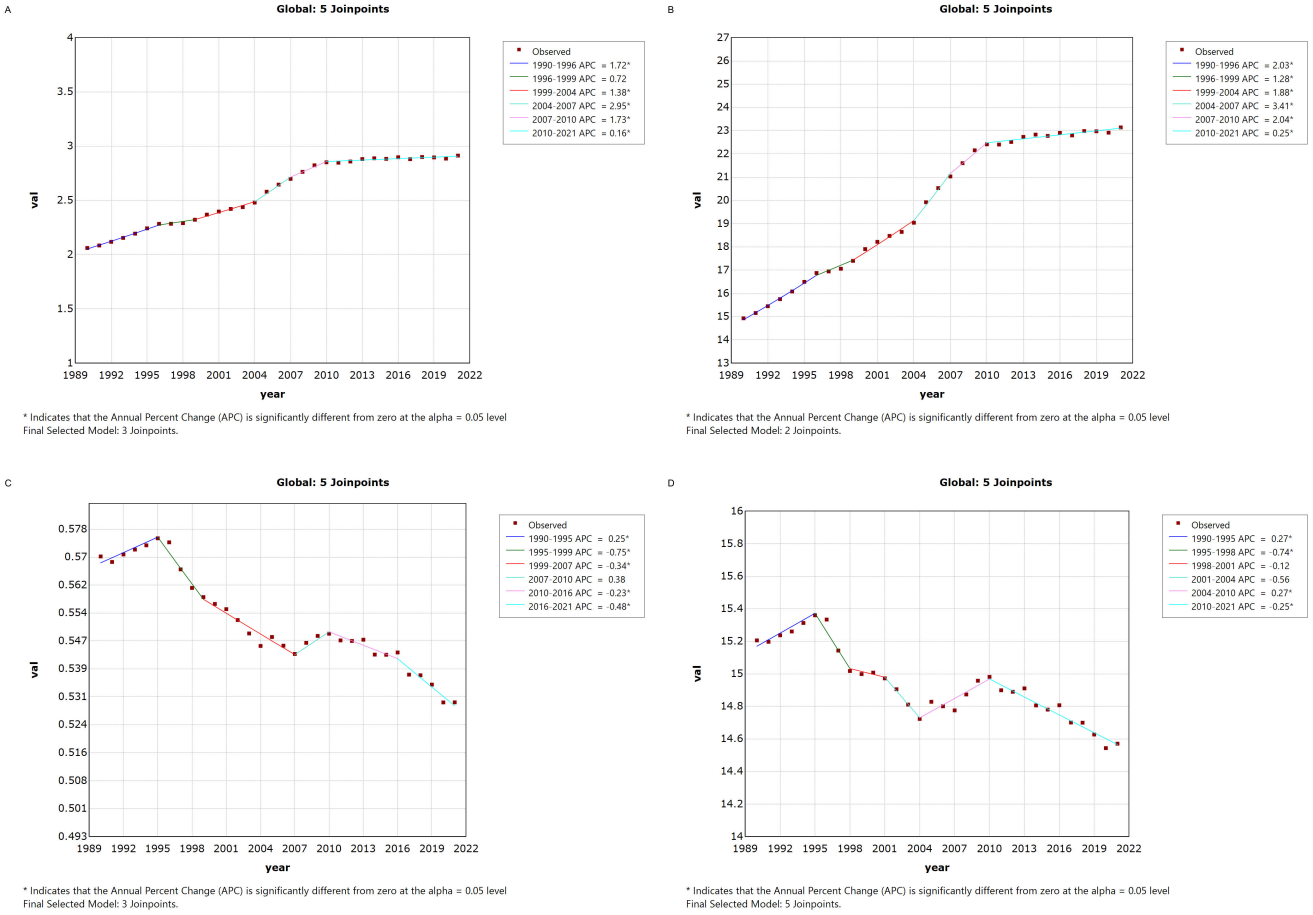
Annual Percentage Change (APC) in Age-Standardized Incidence Rate (ASIR), Age-Standardized Prevalence Rate (ASPR), Age-Standardized Mortality Rate (ASMR), and Age-Standardized Disability-Adjusted Life Year Rate (ASDR) for Thyroid Cancer Globally from 1990 to 2021 (* indicates p < 0.05, denoting significant results). **(A)** ASIR; **(B)** ASPR; **(C)** ASMR; **(D)** ASDR.

**Figure 1** illustrates the Joinpoint regression analysis of the age-standardized incidence rate (ASIR), prevalence rate (ASPR), mortality rate (ASMR), and disability-adjusted life years (ASDR) for thyroid cancer in China from 1990 to 2021. Over this period, the ASIR and ASPR of thyroid cancer in China showed a gradual and significant upward trend, with a positive annual percentage change (APC), whereas the ASMR and ASDR exhibited an overall declining trend, characterized by a negative APC. However, between 2007 and 2010, there was a temporary increase in both ASMR and ASDR, as indicated by a positive APC during this period.Similarly, **Figure 2** depicts the Joinpoint regression analysis for ASIR, ASPR, ASMR, and ASDR of thyroid cancer globally from 1990 to 2021. Globally, the ASIR and ASPR followed a steady and significant upward trajectory, with a positive APC, while ASMR and ASDR showed an overall decreasing trend, with a negative APC. Notably, there were brief periods of rising ASMR and ASDR, such as between 1990–1995 and 2007–2010, where the APC turned positive.

### 3.3 Trends in thyroid cancer burden in China and globally

**Figure 3:**
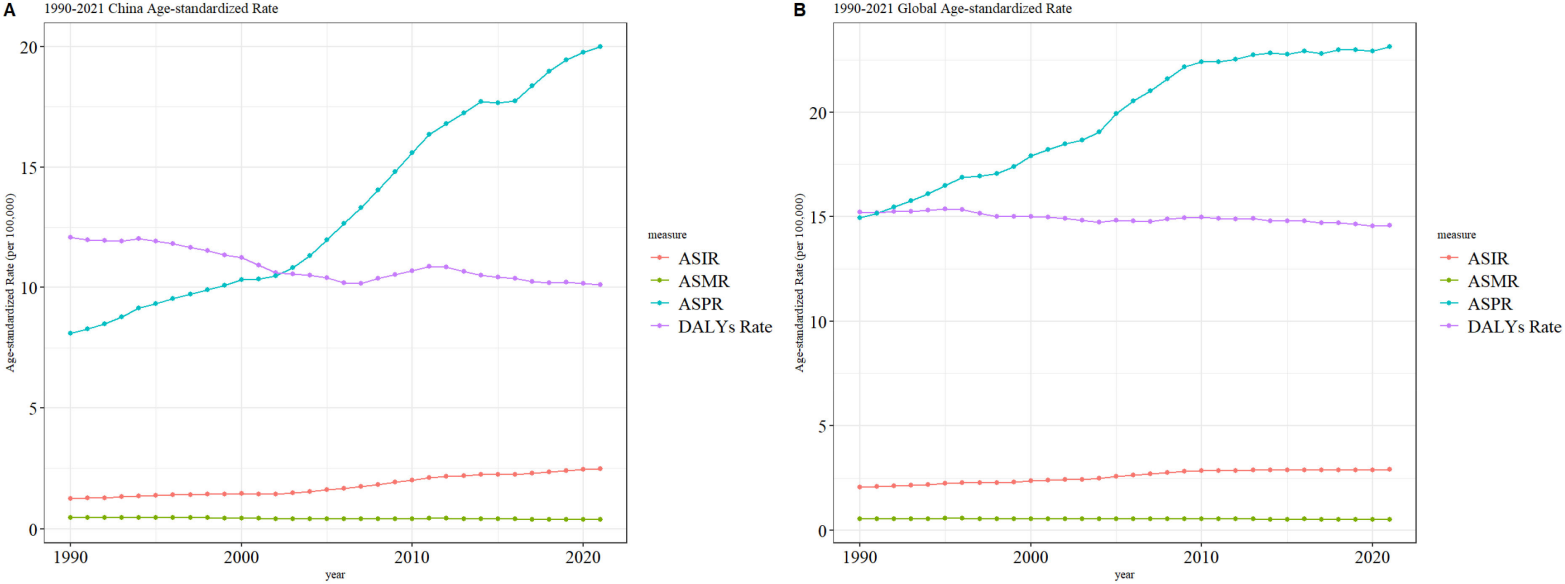
Comparative trends of ASIR, ASPR, ASMR, and ASDR for thyroid cancer in China and Globally, 1990–202 [**(A)** Comparative trends of Age-Standardized Incidence Rate (ASIR), Age-Standardized Prevalence Rate (ASPR), Age-Standardized Mortality Rate (ASMR), and Age-Standardized Death Rate (ASDR) for thyroid Ccancer in China, 1990–2021. **(B)** Comparative trends of Age-Standardized Incidence Rate (ASIR), Age-Standardized Prevalence Rate (ASPR), Age-Standardized Mortality Rate (ASMR), and Age-Standardized Death Rate (ASDR) for thyroid cancer Globally, 1990–2021].

From 1990 to 2021, the age-standardized mortality rate (ASMR) for thyroid cancer in both China and globally remained stable, with no significant fluctuations or changes. However, during the same period, the age-standardized incidence rate (ASIR) and age-standardized prevalence rate (ASPR) of thyroid cancer in both China and globally showed a gradual increase, with the ASPR rising more prominently than the ASIR. The age-standardized disability-adjusted life years (ASDR) for thyroid cancer exhibited an overall downward trend in both China and globally, although the fluctuations in China’s ASDR were more pronounced compared to the global pattern. The specific trends are illustrated in **Figure 3**.

### 3.4 Thyroid cancer burden across different age groups in China and globally, 1990 and 2021

**Figure 4:**
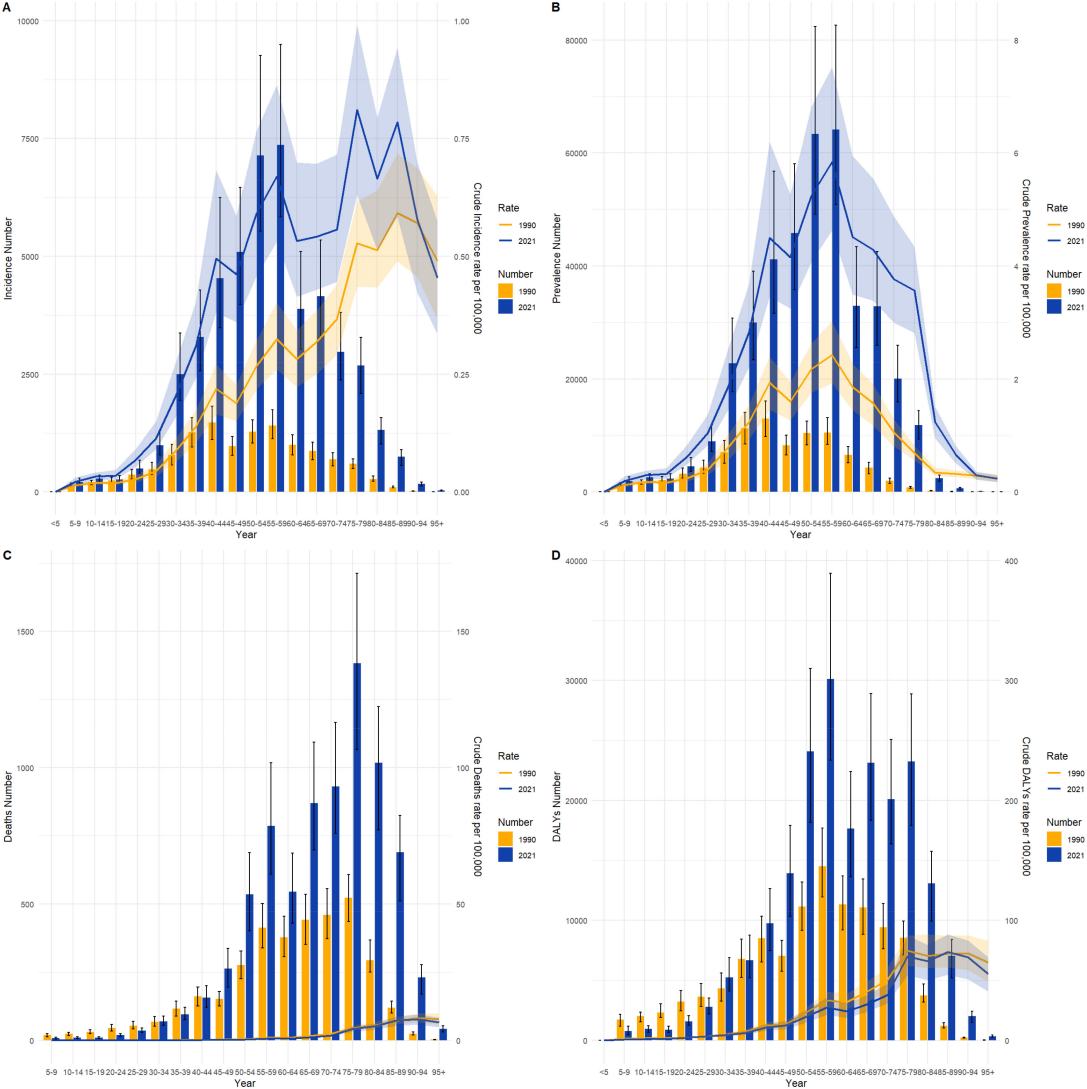
Comparison of thyroid cancer incidence, prevalence, mortality, and DALY counts and crude rates by age group in China, 1990 and 2021. **(A)** Incidence and incidence rate. **(B)** Prevalence and prevalence rate. **(C)** Mortality and mortality rate. **(D)** Disability-Adjusted Life Years (DALY) and DALY rate.

**Figure 5:**
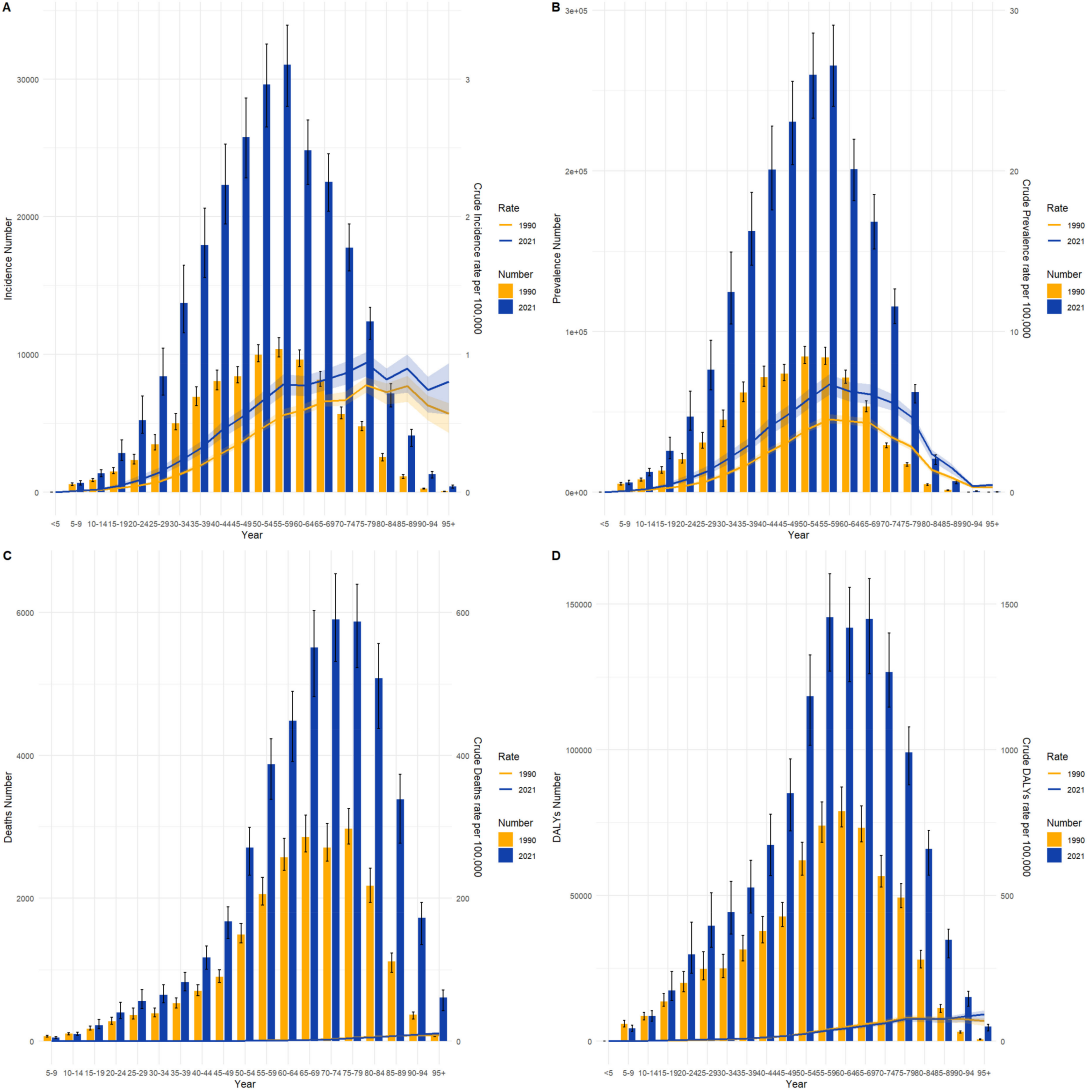
Comparison of thyroid cancer incidence, prevalence, mortality, and DALY counts and crude rates by age group globally, 1990 and 2021. **(A)** Incidence and incidence rate. **(B)** Prevalence and prevalence rate. **(C)** Mortality and mortality rate. **(D)** Disability-Adjusted Life Years (DALY) and DALY rate.

**Figures 4** and **5** compare the incidence, prevalence, mortality, and DALY counts of thyroid cancer, as well as the corresponding crude rates across different age groups in China and globally for 1990 and 2021. In terms of crude rates, both China and global data for 1990 and 2021 show that the crude incidence rate (CIR), crude prevalence rate (CPR), crude mortality rate (CMR), and crude DALY rate (CDR) for thyroid cancer generally increased with age, peaking before declining. The inflection point for CPR occurred between the ages of 55–59, whereas the CIR, CMR, and CDR reached their peak in the 85–89 age range. In terms of counts, the incidence, prevalence, mortality, and DALY counts for thyroid cancer in China and globally for 1990 and 2021 followed a similar trend of rising with age before declining. The highest values for incidence and prevalence counts across different age groups in both China and globally were consistently observed in the 50–54 age range. However, the highest mortality and DALY counts occurred in the 60–64 age range.

### 3.5 Thyroid cancer burden by gender in China and globally, 1990 and 2021

**Figure 6:**
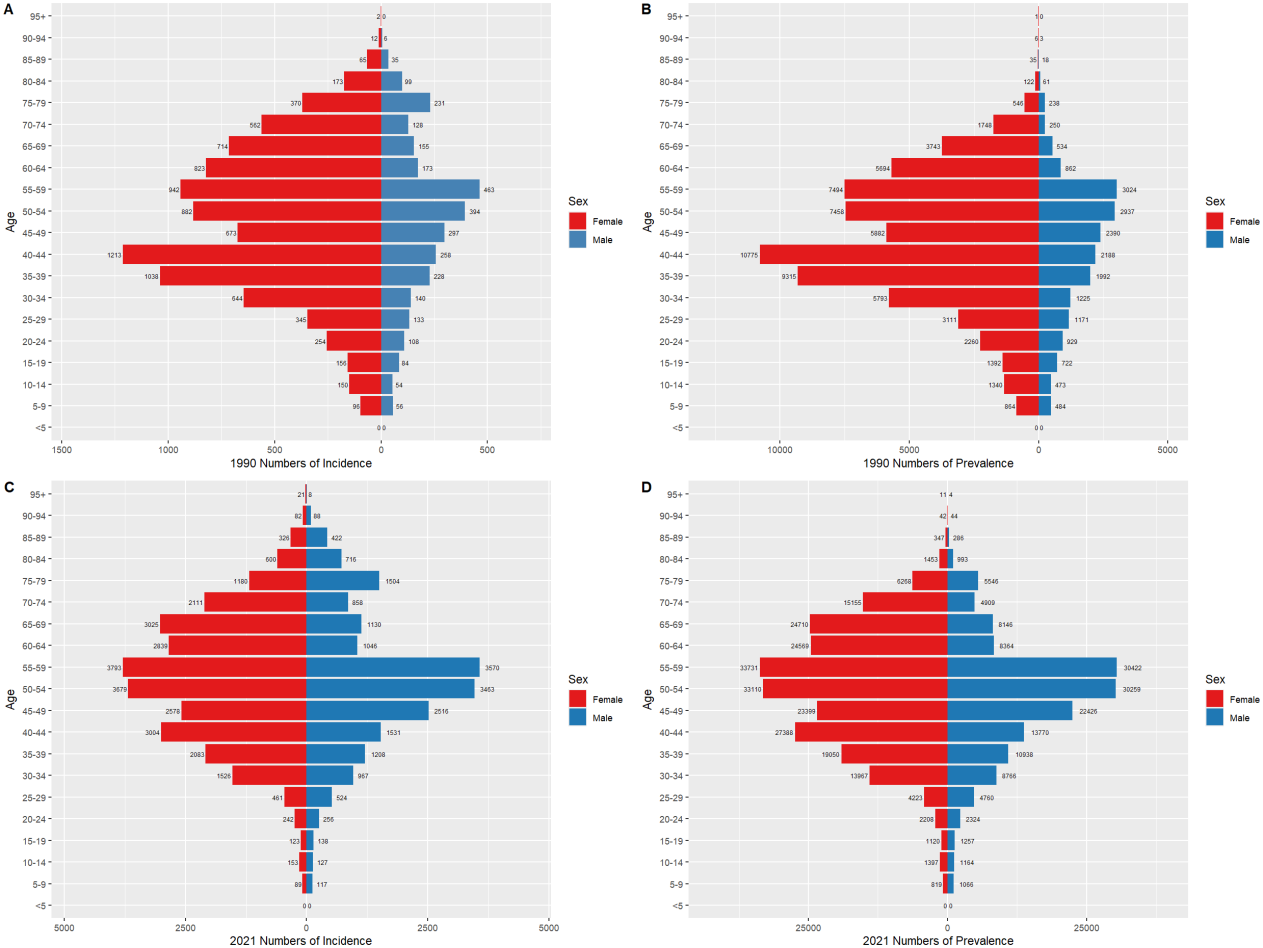
Comparison of thyroid cancer incidence and prevalence counts by age group and gender in China, 1990 and 2021. **(A)** Incidence of new cases in China, 1990. **(B)** Prevalence of cases in China, 1990. **(C)** Incidence of new cases in China, 2021. **(D)** Prevalence of cases in China, 2021.

**Figure 7:**
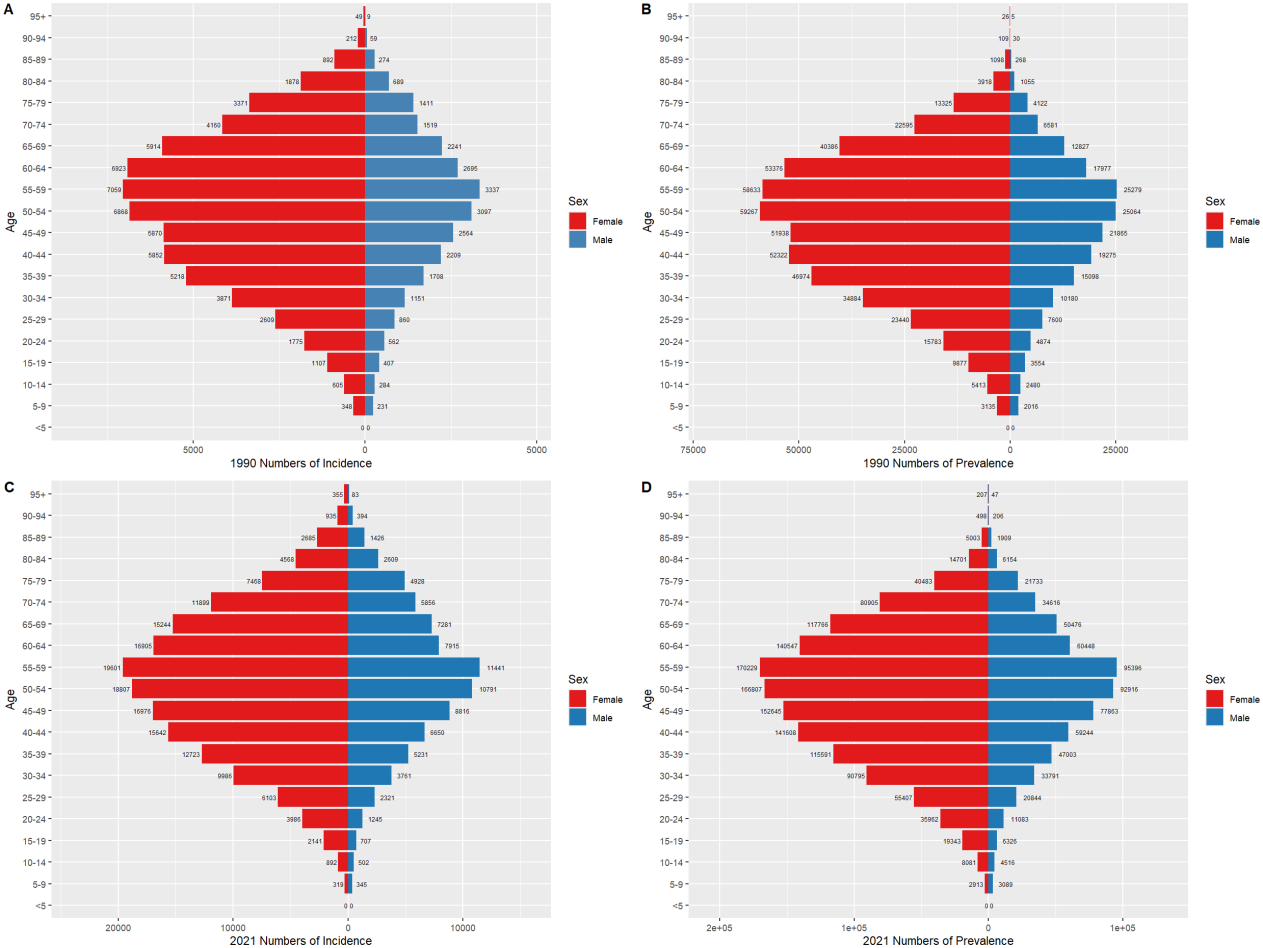
Comparison of thyroid cancer incidence and prevalence counts by age group and gender globally, 1990 and 2021. **(A)** Global incidence, 1990. **(B)** Global prevalence, 1990. **(C)** Global incidence, 2021. **(D)** Global prevalence, 2021.

**Figures 6** and **7** compare the incidence and prevalence counts of thyroid cancer by age group and gender in China and globally for 1990 and 2021. The results indicate that, irrespective of the year (1990 or 2021) and location (China or globally), the incidence and prevalence counts of thyroid cancer were significantly higher in females than in males. However, the peak incidence and prevalence counts for both genders were consistently observed in the 50–59 age range.

### 3.6 Global changes in thyroid cancer prevalence (1990–2021)

**Figure 8:**
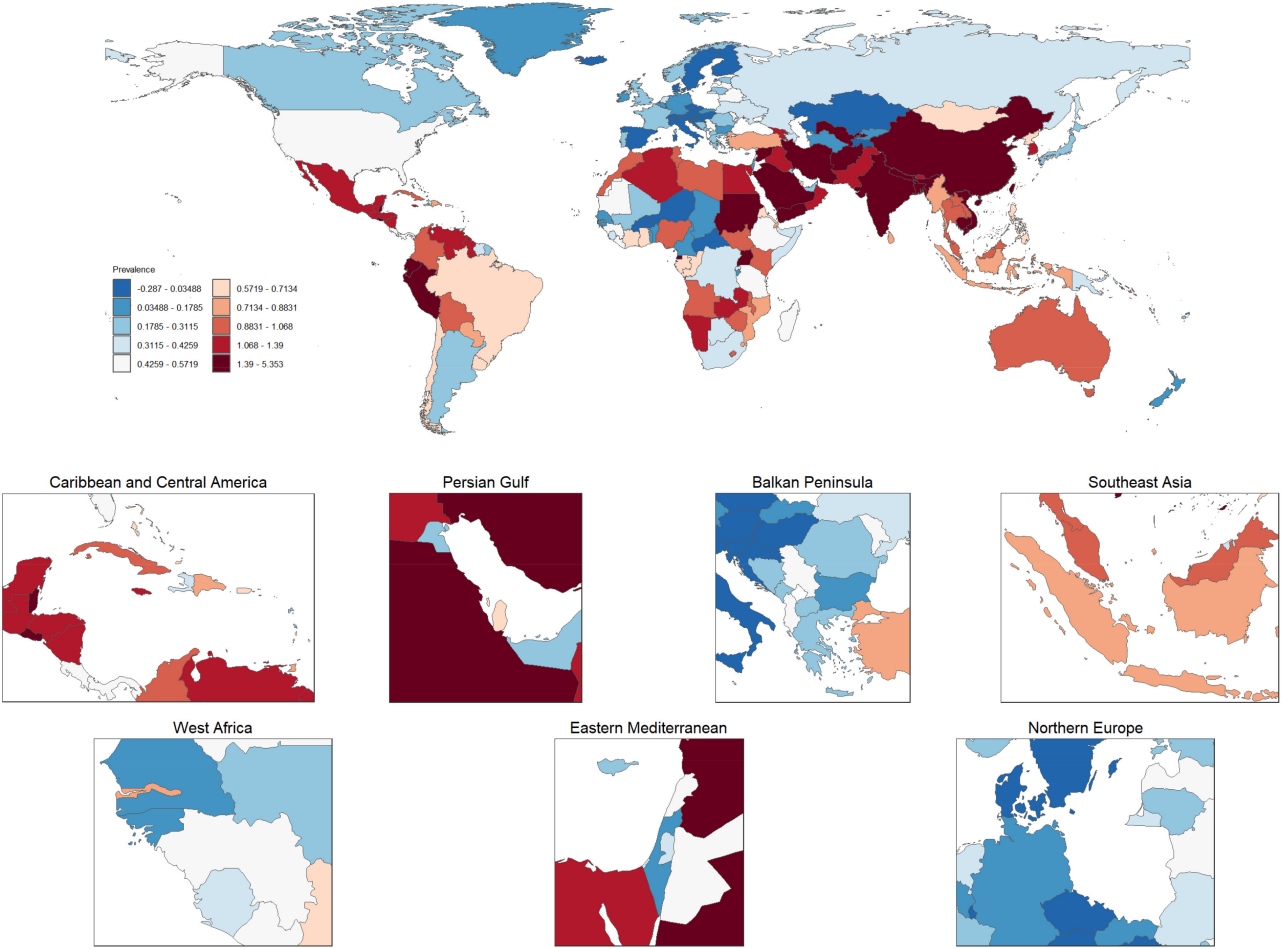
Global and regional distribution of age-standardized thyroid cancer prevalence rates (1990–2021). This figure presents the global distribution of age-standardized thyroid cancer prevalence rates, averaged from 1990 to 2021. The top map illustrates country-level thyroid cancer prevalence, where darker red shades indicate higher prevalence and lighter blue shades represent lower prevalence. The zoomed-in regional maps provide detailed views of specific areas, including the Caribbean and Central America, Persian Gulf, Balkan Peninsula, Southeast Asia, West Africa, and Northern Europe. Variations in prevalence are evident, with regions like China and Southeast Asia showing significantly higher prevalence, while parts of Europe and the Americas display lower rates. These disparities highlight the influence of various factors, such as environmental conditions, healthcare access, and lifestyle differences, in shaping the global thyroid cancer burden.

**Figure 8** illustrates the global distribution of age-standardized prevalence rates of thyroid cancer, averaged over the period from 1990 to 2021. The world map at the top of the figure displays the prevalence rates of thyroid cancer across various countries, with darker red regions representing areas with higher prevalence, while lighter blue regions indicate lower prevalence. Notably, thyroid cancer prevalence is particularly high in China and parts of Southeast Asia, as indicated by the deep red coloring, highlighting significant public health concerns in these regions. In contrast, countries in Northern Europe and North America show much lower prevalence, denoted by lighter blue shades. Below the global map are six zoomed-in regional maps, focusing on the Caribbean and Central America, Persian Gulf, Balkan Peninsula, Southeast Asia, West Africa, and Northern Europe. Each regional map provides a more detailed view of the thyroid cancer prevalence within these areas. For instance, Caribbean and Central America display moderate to high prevalence, with some countries marked in red, indicating particularly elevated rates. Similarly, many countries in the Persian Gulf region also exhibit high prevalence rates. In the Balkan Peninsula and Northern Europe, there is notable variation across countries, with some showing lower prevalence (in blue) and others displaying moderate rates. In the Southeast Asia map, countries like Indonesia have high prevalence rates, while surrounding regions exhibit moderate levels. West Africa, on the other hand, is characterized by lower to moderate prevalence, suggesting that the thyroid cancer burden in this region is relatively light. Overall, this figure highlights the marked global and regional disparities in thyroid cancer prevalence, with the burden being particularly high in parts of Asia and the Middle East, whereas Europe and the Americas generally experience lower to moderate levels. These variations in prevalence may reflect a combination of factors, including environmental exposures, access to healthcare, diagnostic capacities, and lifestyle differences. The findings underscore the urgent need for targeted strategies to alleviate the thyroid cancer burden in high-prevalence regions.

### 3.7 Relationship between the socio-demographic index and the burden of thyroid cancer

**Figure 9:**
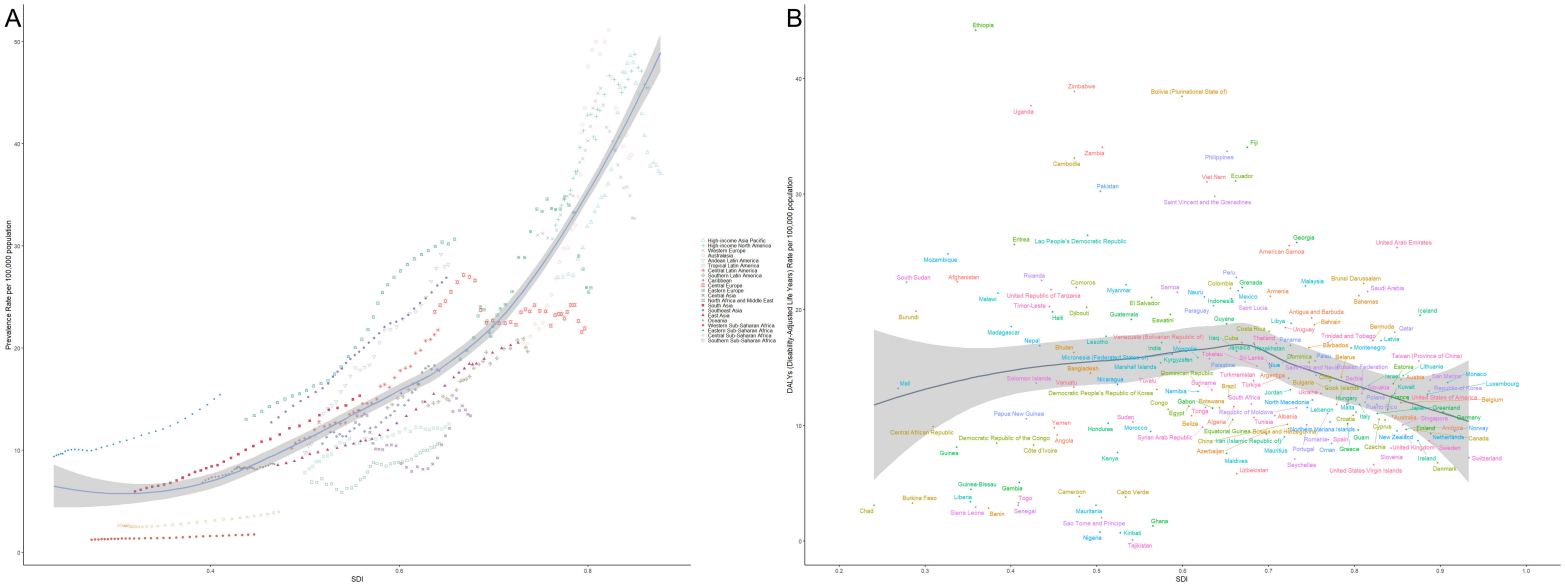
Relationship between thyroid cancer prevalence and burden across different levels of the Socio-Demographic Index (SDI). **(A)** Shows the relationship between thyroid cancer prevalence and SDI across various regions, with prevalence increasing significantly with SDI, particularly in high-income areas such as Asia-Pacific, North America, and Western Europe. **(B)** Illustrates the trend in Disability-Adjusted Life Years (DALYs) attributable to thyroid cancer, with higher-SDI regions showing lower DALY burdens due to better healthcare resources and patient outcomes. Different symbols and colors represent various geographical regions, with data drawn from global statistics across different countries and regions.

**Figure 9** illustrates the global trends in the burden of thyroid cancer across countries and regions in relation to the Socio-Demographic Index (SDI). Panel A shows the prevalence rate, while Panel B depicts the Disability-Adjusted Life Years (DALYs). Different symbols and colors represent various geographical regions. In Panel A, the prevalence of thyroid cancer increases significantly with rising SDI, particularly in high-income regions such as the Asia-Pacific, North America, and Western Europe, where prevalence is markedly higher than in other areas. This suggests that as socioeconomic development progresses, the detection rates of thyroid cancer may improve, potentially due to lifestyle factors or environmental influences. The steeper curve in high-income regions like the Asia-Pacific and North America indicates a more substantial burden of the disease in these areas. In contrast, sub-Saharan Africa, South Asia, and other low- and middle-income regions exhibit relatively lower prevalence rates, likely reflecting limited healthcare resources and lower early detection rates. Panel B presents the trend in DALYs attributable to thyroid cancer across different SDI levels. Unlike the prevalence, the trend in DALYs is more gradual, with higher SDI regions showing lower DALY burdens, which suggests better healthcare access and improved prognosis for thyroid cancer patients. Conversely, in low-SDI regions such as sub-Saharan Africa and South Asia, the DALY burden is relatively higher, indicating potential deficiencies in disease diagnosis and treatment, leading to higher mortality and disability rates. Overall, the figure clearly demonstrates the striking differences in thyroid cancer burden across regions with varying SDI levels. High-SDI regions experience higher prevalence due to advanced diagnostic tools but bear a relatively lower overall disease burden, while low-SDI regions face a heavier burden despite lower prevalence rates, highlighting the ongoing global challenges in managing and treating this disease.

### 3.8 Projections of Thyroid Cancer Burden by Gender in China and globally for the next 15 years

**Figure 10:**
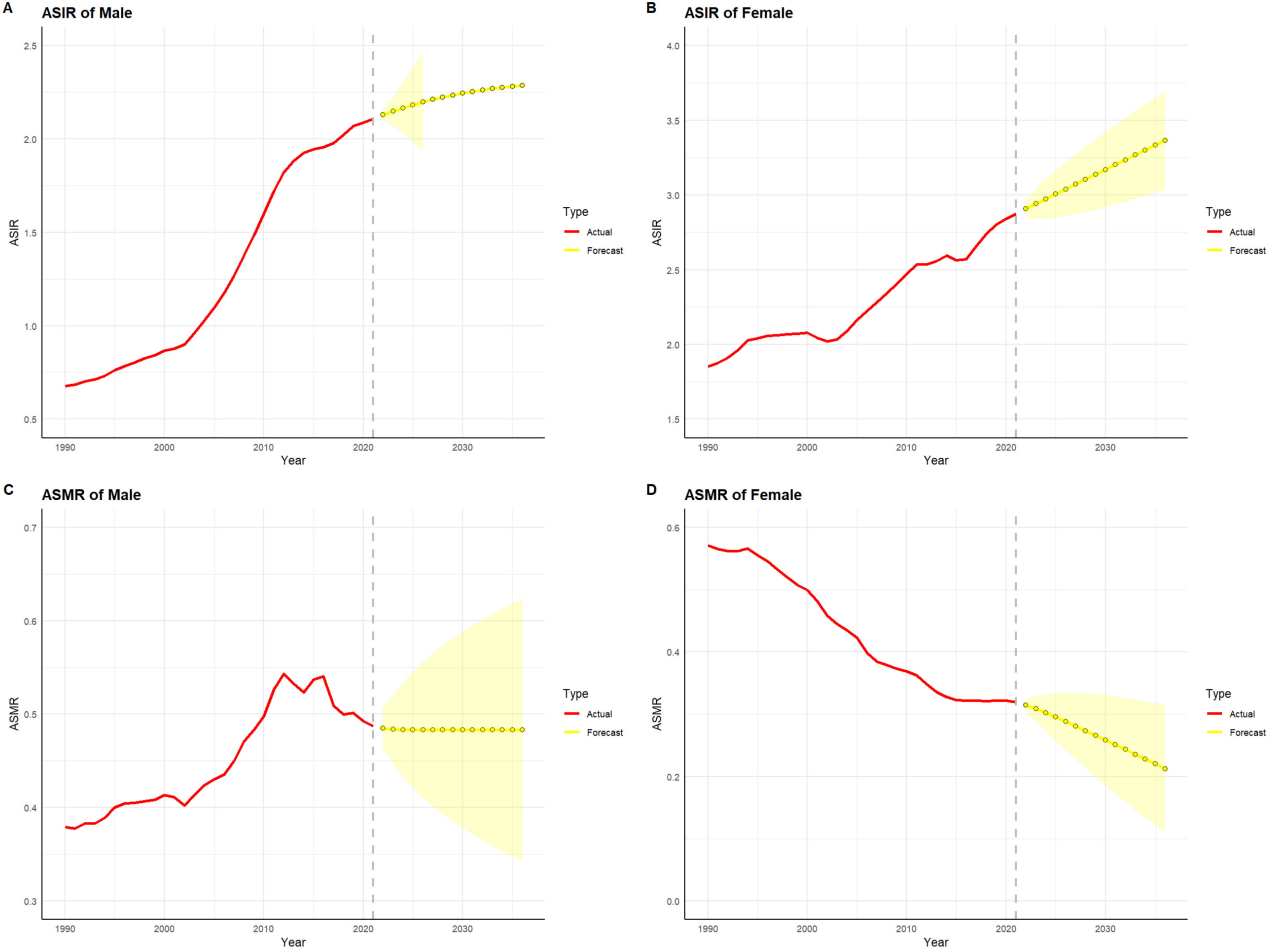
Projected trends in thyroid cancer incidence and mortality rates in China (2022–2036). Red lines represent the observed trends in incidence and mortality from 1990 to 2021 in China, while the yellow dashed lines and shaded areas indicate the projected trends and their 95% confidence intervals. **(A)** Trends in Male Age-Standardized Incidence Rate (ASIR), 1990–2036. **(B)** Trends in Female Age-Standardized Incidence Rate (ASIR), 1990–2036. **(C)** Trends in Male Age-Standardized Mortality Rate (ASMR), 1990–2036. **(D)** Trends in Female Age-Standardized Mortality Rate (ASMR), 1990–2036.

**Figure 11:**
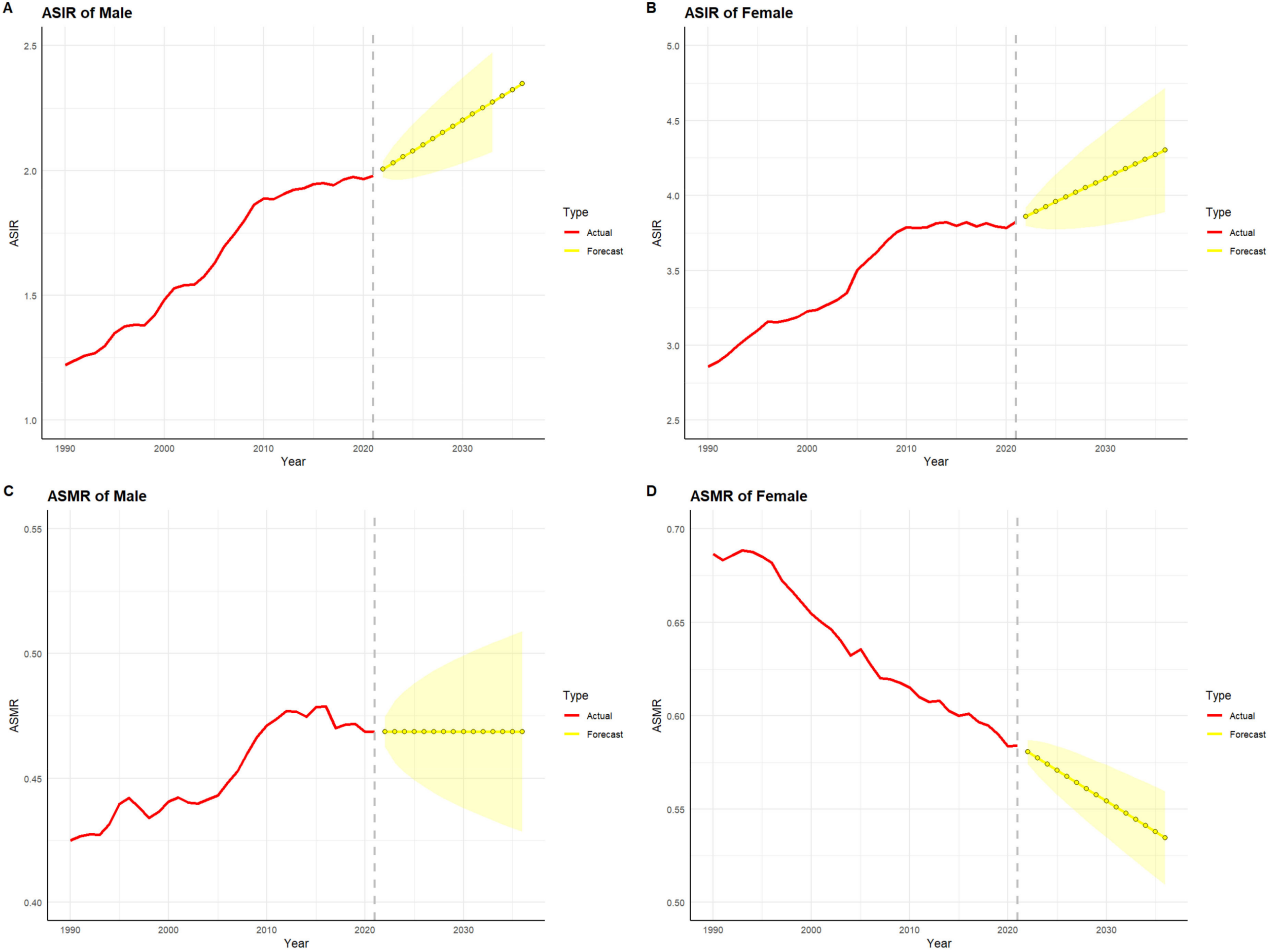
Projected trends in thyroid cancer incidence and mortality rates globally (2022–2036). Red lines represent the observed trends in incidence and mortality from 1990 to 2021 globally, while the yellow dashed lines and shaded areas indicate the projected trends and their 95% confidence intervals. **(A)** Trends in male Age-Standardized Incidence Rate (ASIR), 1990–2036. **(B)** Trends in female Age-Standardized Incidence Rate (ASIR), 1990–2036. **(C)** Trends in male Age-Standardized Mortality Rate (ASMR), 1990–2036. **(D)** Trends in female Age-Standardized Mortality Rate (ASMR), 1990–2036.

**Figures 10** and **11** depict the projected trends in thyroid cancer incidence and mortality rates for males and females in China and globally over the next 15 years, using the ARIMA model. The results indicate that the projected burden of thyroid cancer shows similar trends across genders for both China and the global population. The age-standardized incidence rate (ASIR) for both males and females is expected to gradually increase in China and globally over the next 15 years. The age-standardized mortality rate (ASMR) for male thyroid cancer patients in both China and globally is projected to stabilize, whereas the ASMR for females is anticipated to show a slight downward trend.

## 4 Discussion

Thyroid cancer is one of the most common endocrine malignancies worldwide. While the overall survival rate is relatively high, its rising incidence in many countries, particularly in more developed nations, has made it a growing public health concern. Globally, approximately 560,000 new thyroid cancer cases are diagnosed each year, with a female-to-male incidence ratio of roughly 3:1 (17–19). According to the latest epidemiological data, thyroid cancer can be classified into four major histological types: differentiated thyroid cancer, undifferentiated thyroid cancer, medullary thyroid cancer, and poorly differentiated thyroid cancer. Differentiated thyroid cancers, such as papillary and follicular carcinoma, are the most common and have a relatively favorable prognosis, with a five-year survival rate generally exceeding 90%, and the survival rate for papillary carcinoma approaching 95%. In contrast, undifferentiated thyroid cancers, such as anaplastic carcinoma, have a poor prognosis, with a five-year survival rate typically below 20% and rapid disease progression. Medullary thyroid cancer has a five-year survival rate of approximately 75-80% and is strongly associated with genetic predisposition, with some patients carrying mutations in the RET proto-oncogene. Poorly differentiated thyroid cancer has a five-year survival rate between that of differentiated and undifferentiated types, typically ranging from 40-60%, and its biological behavior lies between these two extremes (20). According to the 2021 Global Cancer Burden report, the annual incidence of thyroid cancer is increasing globally, particularly among women, with notable rises observed in countries such as the United States and South Korea (21). This trend is attributed to improvements in early screening and diagnostic technologies. Furthermore, experts have recommended an active surveillance approach for papillary microcarcinoma. They argue that papillary microcarcinoma often has a relatively favorable prognosis and a slow biological course in many cases, making active monitoring an effective strategy to avoid overtreatment and reduce unnecessary surgical and therapeutic interventions. In recent years, China has updated its guidelines for the diagnosis and treatment of thyroid nodules, emphasizing early screening, fine needle aspiration (FNA) biopsy, and individualized treatment plans (22). These updated strategies, particularly in the management of differentiated thyroid cancer, have further enhanced patient survival rates and quality of life.

The primary risk factors for thyroid cancer include radiation exposure, genetic susceptibility, excessive or deficient iodine intake, and certain environmental factors (6). In recent years, advancements in ultrasound screening and fine-needle aspiration biopsy have significantly increased the detection rate of thyroid cancer, particularly in subclinical early-stage cases (23). However, this surge in early detection has raised concerns about overdiagnosis, especially for papillary thyroid cancer, which often grows slowly and has low clinical aggressiveness (24, 25). Some experts advocate for “active surveillance” rather than immediate surgical intervention for papillary thyroid cancers smaller than 1 cm (20, 26).

This study, based on the GBD 2021 database, evaluates global trends in thyroid cancer incidence, prevalence, mortality, and DALYs over the past 30 years, analyzing the disease burden differences across age and gender. The results reveal that both in 1990 and 2021, the crude incidence rate (CIR), crude prevalence rate (CPR), crude mortality rate (CMR), and crude DALY rate (CDR) of thyroid cancer in China and globally follow a pattern of increasing with age before declining, with the CPR peaking in the 55–59 age group, while the CIR, CMR, and CDR peak later, around 85–89 years of age. Additionally, both in 1990 and 2021, regardless of region, the incidence and prevalence counts of thyroid cancer were significantly higher in females than in males. However, the incidence and prevalence counts peaked in the 50–59 age range for both genders. This phenomenon may be attributed to several factors. First, hormonal fluctuations, particularly the variation in estrogen levels, could influence the proliferation and transformation of thyroid cells, leading to a higher incidence of thyroid cancer in women around the menopausal transition. Second, lifestyle factors associated with social and occupational roles in women may contribute to the development of thyroid cancer, with chronic stress, dietary habits, and exposure to environmental pollutants potentially acting as risk factors. As for the age-related differences, the risk of thyroid cancer increases with age, but in the elderly population over 85 years, the clinical manifestations of certain malignancies may be more subtle due to declining physiological functions, which could result in delayed diagnosis and treatment, thereby influencing mortality and disease burden in this age group. Additionally, the gender disparity in thyroid cancer may be related to genetic factors, immune system differences, and sex-specific physiological characteristics.

Moreover, Zhili Dou et al. (27) conducted a study on the global burden of thyroid cancer using the GBD 2019 database, revealing that the top three countries with the highest thyroid cancer case numbers from 1990 to 2019 remained consistent: the United States (11,689 cases in 1990, 26,270 in 2019), China (10,030 cases in 1990, 39,079 in 2019), and India (5,988 cases in 1990, 23,833 in 2019). China also reported the highest number of thyroid cancer-related deaths globally (3,318 in 1990; 95% UI: 2,861-4,133 and 7,239 in 2019; 95% UI: 6,011-8,476). However, the highest country-specific DALYs for thyroid cancer shifted from China (103,492.62 in 1990; 95% CI: 87,958.27–124,715.04) to India in 2019 (217,465.02; 95% CI: 181,111.87–254,845.52). Zhili Dou’s study highlights the heavy burden of thyroid cancer in China and underscores the effectiveness of its public health policies, complementing the findings of this research.

This study has several strengths: It uses the GBD 2021 dataset to compare the thyroid cancer burden in China and globally from 1990 to 2021; It incorporates data on male thyroid cancer patients, providing a more comprehensive understanding of the thyroid cancer burden across both genders; The ARIMA model was employed to project the thyroid cancer burden for the next 15 years, providing a scientific basis for future public health policy decisions.

Nonetheless, this study has certain limitations: It does not provide a detailed analysis of different thyroid cancer subtypes (e.g., papillary, follicular, anaplastic), which could affect prognosis assessments; It lacks an in-depth exploration of major thyroid cancer risk factors and their dynamic changes, such as radiation exposure and iodine intake; It does not thoroughly examine the relationship between treatment modalities (e.g., surgery, radiotherapy, targeted therapy) and the disease burden; The study does not analyze thyroid cancer burden across countries with different income levels; The Bayesian Age-Period-Cohort Model (BAPC) (28–30) was not utilized to project the thyroid cancer burden for the next 15 years.

In summary, the global burden of thyroid cancer over the past 30 years exhibits significant differences by age and gender. By improving screening strategies, reducing overdiagnosis and overtreatment, and optimizing treatment protocols, there is potential to further alleviate the burden of thyroid cancer and enhance patient quality of life in the future.

## 5 Conclusion

From 1990 to 2021, the incidence and prevalence of thyroid cancer have increased globally, particularly among females and individuals aged 50–64. However, both mortality and DALYs have decreased, reflecting the positive impact of advancements in medical technology and the widespread adoption of early screening. Significant gender disparities persist, with females showing much higher incidence rates than males, but also experiencing a more substantial decline in mortality due to the effectiveness of diagnosis and treatment. As populations continue to age and screening technologies evolve, the burden of thyroid cancer is likely to increase, making it a pressing public health issue that warrants continued attention.

## Data Availability

The original contributions presented in the study are included in the article/supplementary material. Further inquiries can be directed to the corresponding author.
